# Supplementary material for: Behavior Problems and Social Competence in Fragile X Syndrome: A Systematic Review
Source: Genes (Basel). 2022 Jan 30;13(2):280. doi: 10.3390/genes13020280 (PMC8871871; doi:10.3390/genes13020280)
Supplement: Supplementary file 1 [file genes-13-00280-s001.zip › genes-1560172-supplementary - update.pdf]

Supplementary Table S1

Authors/ year	Study aims	Control group	ASD control (scale used)	Sample size (n=)/gender/age	Recruitment	Diagnosis	Methodology/Assessment tools	Developmental factors	Statistics	Findings
Bailey et al., 2000 [105]	Examine differences between boys with FXS and those with ASD in general indicators of development, functional abilities, and behavior.	FXS<br>ASD<br>FXS+ASD<br>TD (reference group)	ECI/SG<br>- The Child Autism Rating Scale (CARS) [166]	FXS (n=31)<br>Boys<br>36-95 months<br>Then it ads 13 boys with FXS+ASD	FOS	GT	S/VM<br>-Behavioral Style Questionnaire (BSQ) [167].<br>-The Batelle Developmental Inventory (BDI)[168].	CA	DS<br>EC<br>Y*	Significant differences were found between boys with FXS and boys with Aut in the personal-social domain, with the formers performing better in this domain. Boys with Aut also scored significantly higher in the behavior domain, reflecting more impairment. Compared to the reference sample, both the FXS and the Aut group scored significantly more withdrawing, and boys with FXS were significantly more active than the reference group. Besides, the group with FXS was significantly more active than the Aut group. In the personal-social domain, boys with comorbid ASD and FXS showed higher impairments than boys with FXS only but lower impairments than those with Aut only. Similarly, the group

										with FXS+Aut was more impaired than the FXS only in behavior measures.
Score=13		2	2	2	0	2	1	2	2	
Kau et al., 2000 [104]	Examine the differences between individuals with FXS and those with DD in four behavior problem areas: social avoidance, hyperactivity or attention problems, difficulties with changes, and irritability.	FXS<br>DD	No	FXS (n=41)<br>Boys<br>3-6 years	SC<br>Chs	GT	S/VM<br>-The Child Behavior Checklist (CBCL) [169, 170].<br>-The Aberrant Behavior Checklist-Community (ABC-C) [171, 172].<br>-The Temperament Survey [173].<br>-The Vineland Adaptive Behavior Scale (VABS) [174].	AM<br>IQM	EC<br>Y*	Boys with FXS showed significantly higher scores on social avoidance behavior but fewer scores on withdrawal behavior. Differences were not found between both groups on attention problems, hyperactivity, or general activity. However, high levels of attention problems were found in both samples. Differences did not appear in the flexibility/rigidity, somatic complaints, anxiety/depression, or irritability scales. However, the boys with FXS showed a significantly higher level of general activity than the DD group when considering maternal characteristics.
Score=13		2	0	2	2	2	1	2	2	
Hessl et al., 2001 [66]	Examine the association between genetic variables and environmental variables in	FXS<br>TD<br>(siblings<br>)	No	FXS (n=120)<br>80 boys/40 girls<br>6-17 years	Ch<br>NW<br>M<br>R<br>MC	GT	S/VM<br>-CBCL [85].<br>-The SCL-90-R [190].<br>-The HOME [175].	SA	DS<br>WR<br>Y*	Internalizing and externalizing problems in boys with FXS were predicted by the effectiveness of educational/therapeutic services and psychological issues in their parents. The quality of the

	behavior problems of individuals with FXS compared to their TD siblings.						-The special Curriculum Opportunity Rating Scale (SCORS) -The Autism Behavior Checklist (ABC) [176].			conditions at home predicted autism symptomatology. In girls, FMRP percentage influenced social withdrawal scores and anxious/depressed behavior.
Score=13		2	0	2	2	2	1	2	2	
Cornish et al., 2001 [101]	Examine the behavior profile on attention and hyperactivity between individuals with FXS, DS, and TD individuals.	FXS DS TD	None	FXS (n=25) Boys 8-15 years	Ch	GT	S/VM -Comprehensive Teacher Rating Scale (ACTeRS)[177]. -CBCL [85].	VIQ-matched CA-matched	CE Y*	Compared to the DS group, the FXS group showed significantly lower scores on attention and higher scores on hyperactivity. Significantly higher scores on attention problems and anxiety were found for the FXS than in individuals with DS. Similar hyperactivity and attention problems were found between TD (EC or EM paired) individuals with inadequate attention and FXS individuals. In contrast, those with DS scored better but well below those with TD with good attention scores.
Score=11		2	0	1	1	2	1	2	2	
Hatton et al., 2002 [22]	Explore the trajectory of behavior problems	None	No	FXS (n=59) 4-12 years boy	MC	GT	S/VM -CBCL [85,178]. -The BSQ [167].	LS (3 years)	DS WC WR	Regarding the clinical concern of problem behaviors, attention, thought, and social problems

	in boys with FXS over three years. Determine the most challenging behavior problems, and discern which variables might predict the behavioral outcomes.						-The CARS [166].			were the ones with the higher percentage. Stability on behavior problems was found all over the three years. Higher autistic symptomatology was significantly associated with greater behavior problems. Higher education of mothers was significantly associated with higher rates on behavior problems as attention, thought, and total scales.
Score=11		0	0	2	2	2	1	2	2	
Rogers et al., 2001 [26]	Examine the behavioral phenotype of young children with FXS compared to young children with AD and DD. Specifically, this study focuses on ASD symptomatology and developmental, including socialization.	FXS AD DD	SG -The Autism Diagnostic Interview -Revised (ADI-R) [179]. -The Autism Diagnostic Observation Schedule (ADOS) [180].	FXS (n=24) 21-48 months	MAP C NW M	GT	S/VM -The VABS [174].	AS	DS CFVSF+ ASD CE Y*	The group with FXS+ASD and the AD group differed from the DD group on social scales with the groups with ASD having higher impairments. The FXS group only did not differ on any adaptive scale from the DD group including socialization.

			-The Diagnostic and Statistical Manual of Mental Disorders -IV [181].							
Score=14		2	2	1	2	2	1	2	2	
Steinhausen et al., 2002 [79]	Examine the behavioral phenotype of four syndromes: Fetal Alcohol Syndrome (FAS), Prader-Willi syndrome (PWS), Tuberous Sclerosis (TS), and FXS.	FAS<br>PWS<br>FXS<br>TS	No	FXS (n=49)<br>Boys<br>5.7-16.10 years	Ch, SC	NM	S/VM<br>-The Developmental Behavior Checklist (DBC) [182, 183].	SA	DS<br>EC<br>Y*	Boys with FXS and those with Fetal Alcohol syndrome showed relatively high frequencies of behavior problems. The FXS scores were the second higher. The most frequently reported behavior problems for boys with FXS were overexcited/impulsive, abusive/swear, irritable, and attention-seeking. Significant differences were found for the six subscales of the DBC, with boys with FXS scoring highest on self-absorbed, communication disturbances, anxious, and autistic relating behaviors. At the same time, individuals with FAS scored higher than those with FXS for disruptive behavior and antisocial. The boys with FXS

										scored significantly higher than those individuals with PWS and Tuberous Sclerosis on disruptive behavior and higher than those with tuberous sclerosis on antisocial behaviors.
Score=11		2	0	2	2	0	1	2	2	
Von-Gontard et al., 2002 [87]	Examine behavior problems and the emotional impact in boys' families with FXS and Spinal Muscular Atrophy.	FXS SMA TD	No	FXS (n=49) Boys 5.7-16.10 years	Ch, MC	NM	S/VM -The CBCL [85]. - The 'Kinder-DIPS' [184]. - F-SOZU questionnaire [185]. - Questionnaire on Resources and Stress [186,187]. - Family Crisis Orientated Personal Evaluation Scale (F-COPES) [188].	SA	DS WR EC Y*	Compared to SMA and TD children, boys with FXS showed more significant behavior problems, particularly externalizing. 73.5% of the subjects with FXS had a comorbid ADHD diagnosis.
Score=11		2	0	2	2	0	1	2	2	
Hatton et al., 2003 [92]	Examine the trajectory of adaptive behavior, including socialization in individuals with	FXS FXS+ASD	SG -The CARS [166].	FXS (n=45) 10 girls/35 boys 12-143 months	MC	GT	S/VM - The VABS [174].	LS (8 years)	DS CFVSF+ ASD Y*	Individuals without ASD comorbidity and higher FMRP protein expression levels showed higher scores on socialization and all over subscales. Increases in

	FXS considering age, gender, ethnicity, maternal education, FMRP expression, and ASD symptomatology.									socialization scores over the assessment points were highest for girls with low ASD symptoms, followed by boys with low ASD symptoms, and then the lowest scores and increases were found for the comorbid FXS+ASD boys. Besides, the lowest scores for comorbid FXS+ASD boys were found for the socialization scale.
Score=14		1	2	2	2	2	1	2	2	
Kau et al., 2004 [46]	Examine the specific characteristics of individuals with FXS in their social behavior profile.	FXS<br>FXS+PDD<br>FXS+Aut<br>DLD+Aut<br>IA	SG<br>-ADI-R [179].	FXS (n=23)<br>FXS+PDD (n=18)<br>Boys<br>Mean age 56.4 months	FOS	GT	S/VM<br>-The CBCL [85,169].<br>-The VABS [174].	*SG	DS<br>CFVSF+<br>ASD<br>EC<br>Y*	The group with comorbid FXS+Aut showed significantly higher scores in problem behavior, with internalizing behaviors contributing to this difference compared to FXS only individuals. Higher scores on withdrawn, attention problems, lethargy/social, and stereotypic behavior were also found for the comorbid group. The highest differences in adaptive domains were found for socialization with comorbid FXS+Aut individuals scoring significantly lower. Comparison with IA boys showed that the comorbid group exhibited a milder profile in autism symptomatology with

										a better performance in reciprocal social interaction, although in behavior problems, the comorbid FXS+Aut group was similar to the DLD+Aut group.
Score=11		2	2	1	0	2	1	1	2	
Kaufmann et al., 2004 [48]	Examine the social behavior profile of individuals with FXS across the ASD comorbidity spectrum from individuals with FXS only through individuals with FXS+PDD to individuals with FXS+ASD. Identify predictors of ASD symptomatology and differences in behavior problems exhibited along the spectrum.	FXS<br>FXS+PDD<br>FXS+Aut	SG<br>-The ADI-R [179].	FXS (n=32)<br>Boys<br>Mean age 4.71 years<br>FXS+PDD (n=10)<br>FXS+Aut (n=14)	FOS	GT	S/VM<br>-The CBCL [85, 169].<br>-The ABC-C [171].<br>-The VABS [174].	NM	DS<br>CFVSF+ASD<br>Y*	An increasing impairment in behavior problems was found for the ASD comorbidity spectrum, with individuals with FXS only being the least impaired and those with comorbid ASD being the most impaired. Items that reflect complex social interaction could discern individuals with FXS+PPD and FXS+ASD from the cohort of individuals with FXS only.
Score=10		1	2	2	0	2	1	0	2	
Budimirovic et al., 2006 [112]	Discern the behavioral characteristics that better contribute to	FXS<br>FXS+ASD	SG<br>-DSM-IV [181].	Cross-sectional assessment<br>FXS (n=32)<br>Boys	FOS	GT	S/VM<br>-The VABS [174].	SA<br>LS	DS<br>CFVSF+ASD<br>Y*	Delayed socialization and social withdrawal correlated with FXS+ASD. Adaptive socialization was the strongest

	the comorbid diagnosis of FXS+ASD			3-8 years Longitudinal FXS (n=19) 3-8 years			-The CBCL [85, 169].			predictor of ASD comorbidity in FXS although withdrawal also predicted it. Items representing social avoidance are the main predictors of ASD inside of scales assessing withdrawal. Adaptive socialization skills were associated with verbal reasoning abilities and thus with FXS+ASD comorbidity. Models combining withdrawal and adaptive socialization could distinguish FXS+ASD groups.
Score=12		1	2	2	0	2	1	2	2	
Hessl et al., 2006 [9]	Examine the association between cortisol reactivity and social behavior profile in individuals with FXS compared to TD individuals.	FXS TD	No	FXS (n=90) 32 girls and 58 boys 6-17 years	NM	GT	S/VM -The ABC [189]. -The CBCL [85]. - The HOME [175]. -The SCL-90-R [190]. -ABC [176] OM -A social task observed and rated	SA	DS WR EC Y*	In comparison to the TD group, boys and girls with FXS had inferior gaze, vocal quality, increased discomfort and task avoidance. Increased cortisol reactivity during the social task was associated with autism symptoms (sensory and social relating). Cortisol decreases were associated with more gaze avoidance and cortisol increases with more eye contact in FXS.
Score= 11		2	0	2	0	2	1	2	2	
Sullivan et al., 2006 [237]	Examine the profile of ADHD symptoms in individuals with	FXS TD	No	FXS (n=63) 7-13 years 6 girls 57 boys	FOS	GT*	S/VM -The CBCL [85, 169, 85].	MA	DS WR Y*	Parents and professors rated between 54-59% of individuals with FXS as meeting diagnostic criteria for ADHD and its

	FXS by describing it, comparing it with a sample of TD individuals, and finding predictors that might influence these behavior problems.						-The Childhood Symptom Inventory-4: Parent Checklist (CSI-PC) [191].<br>-The Adolescent Symptom Inventory-4: Parent Checklist (ASI-PC) [191].<br>-The Childhood Symptom Inventory-4: Teacher Checklist (CSI-TC) [191].<br>-The ASI-4: Teacher Checklist (ASI-TC) [191].<br>-The CARS [166]			subtypes. Scores of ADHD symptoms in the FXS sample were significantly higher than those of the general population and the MA-matched TD peers. Differences in assessments of individuals with FXS between parents and professors were found.
Score=11		2	0	2	0	2	1	2	2	
Hall et al., 2006 [100]	Examine the mutual influences of behavior problems in offspring and family functioning as maternal distress comparing individuals with	FXS<br>TD (siblings)	No	FXS (n=150)<br>56 girls and 94 boys<br>Mean age 10.9 years	Chs<br>IW	GT	S/VM<br>-The CBCL [85].<br>-The Teachers Report Form (TRF) [85].<br>-The SCL-90-R [190].<br>-The Family Environment	AS	WR<br>EME	Children's behavior problems influenced maternal distress, but maternal distress did not impact behavior problems in their offspring with either TD or FXS. Family environment was not associated with behavior problems in individuals with FXS, although it was associated

	FXS to those with TD.						Scale (FES) [192].			with behavior problems in individuals with TD.
Score=13		2	0	2	2	2	1	2	2	
Murphy et al., 2007 [97]	Examining the influence of social-information processing demands on eye-gaze avoidance in individuals with FXS, DS or TD.	FXS<br>DS<br>TD	ECI<br>-DSM-IV [181].<br>-The ABC [176].	FXS (n=15)<br>Mean age 16.5 years<br>13-22 years	R<br>NW<br>M	GT	CT*<br>-Experimental eye-gaze task	SA	WR<br>EC<br>Y*	Individuals with FXS were significantly more eye-gaze avoidance during tasks, although differences were not found regarding proportions of eye contact avoidance between social or non-social tasks. Depending on task difficulty, differences were not found between groups. Age did not influence levels of eye avoidance during tasks.
Score=12		2	2	1	2	2	1	2	2	
Hatton et al., 2009 [98]	Describe the phenotype of girls with FXS examining developmental trajectories and the influence of autistic behavior.	No	S<br>-CARS [166]	FXS (n=15)<br>Girls<br>6months-9 years<br>A subsample of 11 girls was also added for some statistics	MC	GT	S/VM<br>-The BDI [168].<br>Personal-social domain	LS<br>9 years	DS<br>WR	Higher scores on autistic symptomatology were significantly associated with worse outcomes in the personal-social domain over the assessment points.
Score=11		0	1	1	2	2	1	2	2	
Hernandez et al., 2009 [94]	Examine the stability of ASD comorbidity in FXS and find social behaviors and skills	FXS<br>FXS+ASD<br>FXS+<br>PDD	SG<br>-DSM-IV [181].<br>-ADI-R [179].	FXS (n=32)<br>Boys<br>FXS+ASD (n=24)<br>Of those<br>FXS+PDD	FOS	GT	S/VM<br>-The CBCL [85, 168].<br>-The ABC-C [171].	LS<br>(3 years)	WR<br>CFVSF+<br>ASD<br>Y*	The diagnostic of ASD remains relatively stable over the years. Socialization scores and peer relationships differentiated between individuals with FXS

	that could predict ASD comorbidity and severity.	FXS+ AUT	-ADOS-G [180].	(n=10), FXS+AUT (n=14) Mean age at T1 56.6 months			-The VABS [174].			only and those with FXS+ASD (PDD and AUT groups) and correlated with autism severity scores. Over the three years of assessment, scores of the FXS only in socialization skills worsened in comparison to those of the FXS+ASD individuals, which improved.
Score=12		1	2	2	0	2	1	2	2	
Roberts et al., 2009 [107]	Examine the development of social approach behaviors over time, its association with cortisol levels, and its relation with autism symptomatology in individuals with FXS.	FXS FXS+AS DTD	SG -The CARS [166].	FXS (n=33) Boys Mean age 3.99 years	Chs MC Rese arch proje ct	GT	S/VM -The Social Approach Scale (SAS) -The VABS [174].	AS	DS CFVSF+ ASD EC Y*	Differences between boys with FXS only and those with comorbid FXS+ASD were found in cortisol levels, elevated for the comorbid group, and poorer scores on the social approach scale. Higher ASD severity was associated with lower levels of cortisol change between the first hour of assessment and the last one.
Score=15		2	2	2	2	2	1	2	2	
Langthor ne et al., 2012 [82]	Explore the functions of problem behaviors in individuals with FXS and SMS in comparison to a control group of NSID.	FXS SMS NSID	No	FXS (n=34) 5-21 years	Ch	DPG T	S/VM -The ABC-C [189]. -The Questions About Behavioral Functions (QABF) [193].	MA MAB	DS WC WR Y*	Behavior problems for individuals with FXS are less likely to be displayed as attention-maintained behaviors than escape or tangible-maintained. Most individuals with FXS presented 2 or 3 topographical classes of

							-The VABS [194].			problem behaviors (attention, tangible, escape, physical discomfort, or self-stimulation), while only 2.9% exhibited one class. .
Score=13		2	0	2	1	2	1	2	2	
Greenberg et al., 2012 [84]	Examine the relationship between family environment and the behavioral phenotype in individuals with FXS through childhood, adolescence, and adulthood.	* FXS ASD	No	FXS (n=167) 24 females/143 males 6-42 years	Chs MC	DPGT	S/VM -The Five Minute Speech Sample (FMSS) [195]. -CBCL [196]. -Adult Behavior Checklist (ABCL) [197].	SG	DS WR	Maternal criticism was related to externalizing symptoms across all age groups with FXS and criticism with total problems for adolescents with FXS. Higher maternal warmth and more positive remarks were negatively associated with externalizing and total problems in children and adults with FXS. Maternal levels of negative and positive environmental factors did not differ among individuals with FXS or ASD, nor did the effect of these variables on behavior problems in their offspring.
Score=13		2	0	2	2	2	1	2	2	
Smith et al., 2012 [35]	Determine if having a comorbid ASD diagnosis is different regarding the behavioral phenotype of	FXS FXS+ASD ASD	SG -Social Communication Questionnaire	FXS (n=106) 19 girls 87 males Mean age 21.62 years FXS+ASD (n=30)	Chs MC	CDP	S/VM -Scales of Independent Behavior-Revised (SIB-R) [199].	AS	DS CE Y*	Comorbid individuals with FXS+ASD displayed greater impairments in social reciprocity and higher scores on challenging behaviors than individuals of the FXS only

	individuals with FXS only or ASD only.		(SCQ) [198].				- The CBCL [196].			group. Repetitive and challenging behavior levels were also higher than the ones of those with ASD only.
Score=15		2	2	2	2	2	1	2	2	
Wolff et al., 2012 [47]	Examine behavioral differences between individuals with FXS+Aut and individuals with IA in social impairments and restricted, repetitive behaviors.	FXS+ASD FXS IA	SG -The ADOS [200].	FXS+ASD (n=23) FXS (n=27) Boys 3-5 years	MCsR	GT	S/VM -The Repetitive Behavior Scales-Revised (RBS-R) [201].	AS	DS EC(FXS+Aut vs IA) Y*(FXS+Aut vs IA)	Differences were not found in stereotypy, self-injury, and sameness rates between boys with FXS+Aut and boys with Aut only, while compulsive and ritual behaviors levels were lower for the FXS+Aut group. Gaze integration, Quality of Social Overtures, Social smile, Facial expression, and joint attention were less impaired in the comorbid FXS+Aut group. The radar graph of the repetitive behavior scale shows a less impaired picture for individuals with FXS only.
Score=13		2	2	1	2	2	1	2	1	
Klaiman et al., 2014 [91]	Examine the trajectories of adaptive behavior in individuals with FXS compared to individuals with TD.	FXS TD	No	FXS (n=275) 89 females/189 males 2-18 years	MC Ch	GT	S/VM -The VABS [174].	LS (12 years)	DS WC EC Y*	In comparison to other adaptive behaviors, socialization scores seem to be an area of strength in boys, with FXS decreasing the least over the years. Compared to TD boys, socialization scores are lower for both girls and boys with FXS.

Score=13		2	0	2	2	2	1	2	2	
Russo-Ponsaran et al., 2014 [108]	Explore the social information processing skills of girls with FXS compared to girls with TD MA-matched on a social task, determine the functioning and explore if autistic symptomatology is associated with performance on social information processing.	FXS<br>TD	*SG<br>-The SCQ [202].<br>-The ADOS [203].	FXS (n=11)<br>7-18 years<br>girls	SC	DPGT	IQ	VMA	DS<br>WC<br>Y*	Girls with FXS were found to reach lower scores in social information processing at the early stages (as identifying problems) than TD girls. They also scored at a lower level for the latter stages as generating competent goals or competent first solutions. Autism symptomatology was associated with competent goal generation.
Score=12		2	2	0	1	2	1	2	2	
Thurman et al., 2014 [73]	Determine the profile of psychiatric symptoms in a sample of boys with FXS and compare its profile with the profile of individuals with ASD.	FXS<br>ASD	S<br>-The ADOS [180].	FXS (n=41)<br>4-10 years<br>Boys	FOS	DPGT	S/VM<br>-The Anxiety Depression and Mood Scale (ADAMS) [204].	CA	DS<br>WC<br>Y*	Hyperactive/manic behaviors and general anxiety were significantly more common in the group with FXS. Social avoidance and general anxiety were associated in boys with FXS more robustly than in the group with ASD.
Score=12		2	1	2	0	2	1	2	2	
Talisa et al., 2014	Examine differences between	FXS	SG<br>IQ	FXS (n=177)<br>3-11 years	MCh	PR	IQ	SG	DS<br>WR	High percentages of attention problems,

[63]	comorbid syndrome as ASD or Anxiety or a combination in the FXS FM phenotype and see if profiles differ from FXS only individuals. Compare differences between age groups.	FXS+ASD<br>FXS+Anx<br>FXS+ASD+Anx		Boys<br>FXS+ASD, FXS+Anx and FXS+ASD+Anx<br>3-11 years<br>112 individuals with FXS, FXS+ASD, FXS+Anx and FXS+ASD+Anx<br>>11 years						hyperactivity/impulsivity, autism and anxiety were found in the sample in both children and adolescents/adults. The sample with comorbid FXS+ASD+Anx showed the highest prevalence of attention problems, hyperactivity/impulsivity, self-injurious behavior and aggressiveness. While in groups of FXS without anxiety (with or without ASD) this scores were lower. Depression and seizures differences were only found in the adolescent/adult sample.
Score=11		1	2	2	2	0	0	2	2	
Rice et al., 2015 [83]	Examine the developmental trajectories of aggression.	FXS<br>DS<br>PW<br>WS	None	FXS (n=63)<br><19 years<br>>19 years	NM	States *that syndrome origins were established through medi	S/VM<br>- The DBC [205]	LS<br>SG	WR	Verbal aggression scores were not significantly associated with age for any group. Physical aggression scores declined over the years for DS, FXS, and WS until 19 years. After this age, physical aggression is not associated with age for FXS or DS.

						cal and genet ic histo ry, and in case of not being sure, other meth ods were follo wed.				
Score=11		2	0	2	0	2	1	2	2	
Hall et al., 2016 [80]	Examine the prevalence, frequency, and severity of problem behaviors in adolescent boys with FXS.	Mixed etiology ID FXS	No	FXS (n=85) Boys 11-18 years	Chs, socia l medi a	NM	S/VM -The Functional Analysis Screen Tool (FAST) [206].	CA SA	DS EC Y*	Boys with FXS showed significantly more frequently self-injurious behavior, more prevalent stereotyped behavior, and aggression was less severe than for the group of boys with mixed-etiology ID. Aggression scores and property destruction correlated with each other in both syndromes. In the group

										with FXS frequency of self-injury decreased with age.
Score=11		2	0	2	2	0	1	2	2	
Smith et al., 2016 [86]	Describe developmental trajectories of behavior problems, psychological symptoms, and ASD symptomatology in individuals with FXS.	None	No	FXS (n=147) 12-48 years at T1 27 females/120 males	MC Chs	DPG T	S/VM - SIB-R [199]. -The CBCL [196]. -The Center for Epidemiological Studies Depression Scale (CES-D)[207]. -The FMSS [195] -The SCQ [198].	LS	WR	Regarding behavior trajectories, increasing age was significantly associated with reduced internalizing symptomatology. Fewer behavior problems appeared in females and of older age. Regarding environmental variables that could affect behavior problems within families, warmth increases were associated with decreases in behavior problems. The higher criticism levels mothers showed between families, the more severe behavior problems individuals with FXS exhibited. Besides, higher levels of maternal depressive symptoms and criticism were associated with higher externalizing behaviors between families. Females at older ages exhibited significantly fewer autism symptoms. Higher warmth between families was also associated with fewer autism symptoms.

Score=11		0	0	2	2	2	1	2	2	
Oakes et al., (2016) [42]	Examine the repetitive behaviors profile of individuals with FXS considering inter-correlations and predictive factors as anxiety, nonverbal cognition, and ASD symptoms that could influence this kind of behavior problems.	No	S<br>-The ADOS [180].	FXS (n=39)<br>Boys<br>6-10 years	R, WS, NWP, flyers at parent meetings	GT	S/VM<br>-The RBS-R [201].<br>-The ADAMS [204].	As a group	DS<br>WR	The most problematic behaviors were restricted interests (with fascination/preoccupation with a subject or activity and strongly attached to an object as the most frequently reported items in the moderate to severe problems) and sensory-motor behaviors (with hand/finger stereotypies and sensory difficulties being the most frequently reported as severe). Self-injury behavior problems were the less severe problems. Anxiety scores correlated positively and significantly with restricted interests, compulsive behavior, and ritualistic/sameness. And ASD social-affective symptomatology was positively associated with restricted interests.
Score=10		**0**	**1**	**2**	**2**	**2**	**1**	**0**	**2**	
Zhu et al., 2016 [106]	Examine the adaptive behavior of individuals with FXS, comparing it to individuals with DS and TD individuals	FXS<br>DS<br>TD	ECI	FXS (n=18)<br>Boys<br>40-167 months	SC<br>S	GT	S/VM<br>-The Infants-Junior Middle School Students' Social life Abilities Scale [208].	MA-matched<br>CA-matched	DS<br>EC<br>Y*	Socialization scores were significantly lower for the boys with FXS. Compared to TD boys matched by CA, socialization scores of boys with FXS were significantly lower, while when comparing boys with FXS to

	matched by CA and MA.									those with TD MA-matched, differences in socialization were not found.
Score=14		2	2	1	2	2	1	2	2	
Caravella et al., 2017 [99]	Examine the developmental trajectories on adaptive behavior comparing infants with FXS, TD, and siblings of individuals with ASD.	FXS TD Infant siblings of children diagnosed with autism (ASIBS)	S/ SG -The Autism Observation Scale for Infants (AOSI) [209]. -The ADOS-2 [210].	FXS (n=25) Boys 6-24 months Of those 11 were comorbid with ASD	R, Chs Social media groups, MC	GT	S/VM -The VABS-II [211].	LS	WR CFVSF+ ASD CE Y*	Compared to the ASIBS and individuals with TD, infants with FXS showed lower socialization scores at nine months. These differences become greater until 24 months, the last study time point. Girls with FXS showed higher socialization abilities than boys at nine months, with girls gaining skills faster. Differences in communication between individuals with only FXS and those with comorbid ASD were found.
Score=13		2	1	0	2	2	1	2	2	
Kaufmann et al., 2017 [111]	Determine how ASD comorbidity influences the behavioral phenotype of individuals with FXS.	FXS FXS+ASD	SG -The SCQ [202]. -The DSM-5 [147]. -The DSM-IV [181].	FXS (n=713) 155 females/ 558 males 0-24 years FXS+ASD (n=237) 0-23 years	MC	GT	S/VM -The ABC [189].	SG	CFVSF+ ASD Y*	Higher proportions of behavior problems were found in individuals with comorbid ASD, including attention problems, hyperactivity, hypersensitivity/over-activity, irritability/aggressive behavior, and perseverative/obsessive-compulsive behaviors. While fewer differences were found for anxiety, only differentiating

										older samples with the comorbid sample showed greater anxiety problems. No differences were found for mood swings/depression.
Score=14		1	2	2	2	2	1	2	2	
Reisinger and Roberts, 2017 [34]	Explore the relationship between social skills and chronological age in boys with FXS compared to boys with TD. And discern how autism and anxiety symptoms are related to social skills when controlled by adaptive behavior.	FXS<br>TD	No	FXS<br>64 boys<br>3-7 years (cross-sectional comparisons)<br>102 boys<br>3-14 years (assessing predictors of social skills)	R, Chs, MC and ongoing studies	GT	S/VM<br>-The CBCL [85,196].<br>-The preschool and elementary version of the Social Skills Rating System (SSRS) [212, 213].<br>-The VABS [174].<br>-The CARS [166].	CA	EC<br>Y*	With age, individuals with FXS and those with TD increased their scores in social skills, although the group with FXS reached significantly lower levels of social skills. Individuals with low levels of ASD symptomatology showed more significant improvements in their social skills as they got older compared to those with a medium level who reached their higher social skills at younger ages showing small increases after that, or those with high levels of ASD who, besides scored lower at earlier stages showed minimal improvements in social skills. In the case of anxiety, boys with FXS with high levels of anxiety showed decreases in self-control as they got older compared to those with medium and low levels

										who showed an increase with age.
Score=13		2	0	2	2	2	1	2	2	
Richards et al., 2017 [102]	Examine the behavioral phenotype of Phelan-McDermid syndrome compared to individuals with FXS, ASD, and DS.	PMDS<br>DS<br>ASD<br>FXS	No	FXS (n=30)<br>Boys<br>6-39 years	Ch	CDP	S/VM<br>-The Activity Questionnaire (TAQ) [214].<br>-The Repetitive Behaviour Questionnaire (RBQ) [215].<br>- SCQ-L [216].	CA	DS<br>EC<br>Y*	Boys with FXS scored significantly lower in over-activity, impulsivity, and total activity scale than those with ASD and significantly higher in the same variables than those with DS. Similarly, boys with FXS scored significantly lower than those with ASD on the insistence on sameness and stereotyped behavior, scoring lower than those with PMD and significantly higher than those with DS. Boys with FXS did not differ from the other groups on compulsive behaviors.
Score=12		2	0	2	1	2	1	2	2	
Martin et al., 2017 [113]	Examine the ability to indicate a communication breakdown in individuals with FXS, DS, and ASD.	FXS<br>FXS+ASD<br>ASD<br>DS<br>TD	SG<br>-The ADOS [217].	FXS (n=38)<br>27 girls/11 boys<br>5.6-16.3 years	R,<br>Ch,<br>MC	NM	OM*<br>Noncomprehension signaling task	S	EC<br>CFVSF+<br>ASD<br>Y*	Boys with comorbid FXS+ASD and boys in the DS group made significantly fewer signals of non-comprehension overall than boys with ASD and TD. Boys with FXS+ASD indeed produced fewer signals of non-comprehension than those with FXS only. Depending on the context, the number of non-

										comprehension signals varied with comorbid FXS+ASD boys signaling fewer than FXS only boys in unfamiliar and ambiguous conditions. Girls with FXS+ASD and girls with DS produced fewer signals of non-comprehension than TD girls. In ambiguous and incompatible conditions, girls with FXS+ASD and DS made fewer signals than TD girls.
Score=12		2	2	2	2	0	1	2	2	
Tonnsen et al., 2017 [109]	Observe trajectories of stranger anxiety in individuals with FXS and TD longitudinally and establish comparisons with parent ratings of anxiety, withdrawal, and autism symptoms.	FXS<br>TD	S<br>-The CARS [166].	FXS (n=46)<br>12 girls/34 boys<br>Mean age 38.85 months	Othe r studi es Chs	DPG T	S/VM<br>-The CBCL [218].<br>-The Laboratory Temperament Assessment Batery (Lab-TAB) [219].	LS S	EC Y*	Over the years, distress vocalizations remained stable while there was an increase in escape behavior for individuals with FXS and TD. Individuals with FXS exhibited fewer facial expressions of fear altogether, tending to increase over the years. In the group with FXS, lower mental age was associated with higher ASD symptoms, escape behaviors, and distress vocalizations. The higher the withdrawal scores, the higher the autistic and anxiety symptoms the group showed. Medium and elevated withdrawal scores were

										associated with stability in distress vocalizations over the years, while inferior levels of withdrawal were related to decreases in distress vocalizations.
Score=14		2	1	2	2	2	1	2	2	
Warren et al., 2017 [93]	Examine the relationship between parenting and the development of adaptive behavior.	FXS FXS+ASD	SG S -The CARS [166].	FXS (n=55) 11 girls/44 boys Between 2.84-9.38 years on average at T1 Of those 18 were classified as comorbid FXS+ASD	R, advertisements at conventions, networking with community	NM	OM -Observation of maternal interactions with their offspring.	LS	WR CFVSF+ASD	There was an increase in socialization scores for individuals with FXS. Those increases were higher for the group without ASD comorbidity and lower for those with ASD comorbidity. Between 80 and 100 months, rates of increase in socialization scores slowed, with many children declining at these stages. Maternal responsivity has a positive influence on socialization scores. Maternal behavior management did not show any influence on adaptive trajectories.
Score=12		1	2	2	2	0	1	2	2	
Crawford et al., 2018 [88]	Examine differences between individuals with and without elevated levels of autism in impulsive	FXS+ASD FXS-ASD	SG -The SCQ [202].	-FXS-ASD (n=37) -FXS+ASD (n=32)	Ch	NM	S/VM -TAQ [214, 220]. -The RBQ [215].	LS SG 8 years	WR	Over time, individuals with FXS with low autism symptoms decreased in their levels of impulsivity and repetitive behaviors. Individuals of the FXS+ASD group scored higher

	and repetitive behaviors. Examine developmental trajectories and links between overactivity and impulsivity with repetitive behaviors.			Mean age at T1=16.32/ 18.43 years<br>T3= 23.76/25.45 years<br>All boys						on the overactivity scale at T1 and T2 and on the impulsivity scale at T3. Over the 8 years overactivity and impulsive scores did not change in both groups. Overactivity and impulsivity scores were not associated with repetitive behaviors in any group.
Score=11		1	2	2	1	0	1	2	2	
Del Hoyo-Soriano et al. (2018) [95]		None	No	-16 FXS Females<br>10-15 years old	NW<br>M<br>IW<br>R	DPG<br>T<br>GT	S/VM<br>-The SCL-90-R [190].<br>-The CBCL [196].	LS<br>3-years	DS<br>WR	The level of withdrawal and anxious/depressed levels was associated with the ratio of affected total chromosomes but not with FMRP levels. Severity of mother symptoms and reciprocated closeness of the mother with withdrawn behavior with higher reciprocated closeness being associated with lower withdrawn in the offspring and higher symptoms in the mother were positively associated with higher withdrawn scores. A trend was found for higher reciprocated closeness and lower anxious depressed behavior in offspring. Scores in both withdrawn and

										anxious/depressed were stable over the 3 years.
Score=10		0	0	1	2	2	1	2	2	
Crawford et al., 2019 [96]	The authors examined the socio-behavioral phenotypes of individuals with FXS, RTS, CdLS, and DS, considering adults' familiarity and interaction environments for social anxiety and social motivation scores.	FXS<br>RTS<br>CdLS<br>DS	No	FXS (n=20)<br>Boys<br>Mean age: 23.68 years<br>>= 11 years.	SC	CDP	IQ<br>-Social Task<br>-The Social Anxiety and Motivation Rating Scale (SAMS).<br>-The SCQ [202].	SA	Y*	Boys with FXS exhibited higher social anxiety than individuals with DS but comparable levels of social motivation. Increasing age was associated with decreasing social anxiety in the FXS group. The higher the social anxiety, the lower the social motivation scores for individuals with FXS.
Score=10		2	0	1	1	2	1	1	2	
Crawford et al., 2019 [81]	Over eight years, examine the prevalence, frequency, and associated risk markers of self-injurious behaviors in individuals with FXS.	No	No	FXS (n=79)<br>Boys<br>6-47 years at T1	SC	CDP (pediatrician or geneticist)	S/VM<br>-The Challenging Behavior Questionnaire [221].<br>-TAQ [214].<br>-The SCQ [202].	LS<br>(8 years)	DS<br>WR	Persistence rates for self-injurious and aggressive behaviors were 77% and 69%. Repetitive behavior scores at T1 predicted continuing self-injurious behavior, while impulsivity predicted persistent aggressive behavior. Over-activity, impulsivity, and age were associated with continuous aggressive behavior.

Score=10		0	0	2	1	2	1	2	2	
Chromik et al., 2019 [236]	Examine the relation between variables associated with ADHD and social functioning in a cohort sample and in a longitudinal sample to find predictors of functioning.	No	No	FXS (n=73)<br>15-25 years<br>40 boys, 33 girls	R<br>PA<br>IW	GT	S/VM<br>-The Attention Deficit/Hyperactivity Disorder-Test [222].<br>-The ABC-C [223].<br>-The CBCL [85].<br>-The VABS [174].<br>-The ADOS [180].	LS	WR	Some ADHD symptoms could predict social functioning on the second time assessment eight years later from the first. In case of females, higher hyperactivity scores at T1 predicted higher social problems. For males, higher socialization scores predicted higher socialization scores and higher hyperactivity contributed to lower socialization scores at T2. In the cohort sample, ADHD symptoms and hyperactivity were associated with social problems and socialization scores.
Score=11		0	0	2	2	2	1	2	2	
Roberts et al., 2019 [89]	Examine the appearance of social anxiety and the developmental trajectory over time in individuals with FXS.	FXS<br>TD	No	FXS (n=191)<br>Boys<br>4-72 months<br>10-25 years	R, past studiessocial media, colleagues, Ch	GT	-The Social Avoidance Scale (SAS) [107,126,].	CA	DS<br>EC<br>Y*<br>OR	81% of boys with FXS exhibited social avoidance appearing at early infancy, worsening through childhood, and stabilizing in adolescents and adults (until 25 years).

Score=13		2	0	2	2	2	1	2	2	
DaWalt et al., 2019 [110]	Explore the differences in friendships, social participation, recreational activities, and family networks between individuals with FXS and those with AD in adolescence and adulthood.	FXS<br>AD	ECI<br>-The ADI-R [179].	FXS (n=81)<br>12-21 years<br>>22 years	MC<br>Ch	DPP<br>C	S/VM<br>MQ<br>-Social participation based on the National Survey of Families and Households [224].<br>-The ADI-R [179].<br>-Zarit Burden Interview [225].	SG<br>S	WR<br>EC<br>Y*	In comparison to individuals with AD, those with FXS had more friends and impacted less the family's social development. In both comparison groups, there was a difference between the adolescent stage and the adult with adolescents being less with friends and neighbors and spending more time exercising than adults.
Score=15		2	2	2	2	2	1	2	2	
Ellis et al., 2020 [90]	Describe the sociability profiles of individuals with FXS, CdLS and RTS taking into consideration both autism and developmental trajectories	FXS<br>CdLS<br>RTS	No	FXS (n=36)<br>Boys<br>Mean age 15.24 years<br>2-46 years	SC<br>Ch	CDP	S/VM<br>-The Child sociability Rating Scale [226-228-235].<br>- The ADOS-2 [210].	AS	CE<br>Y*	In comparison with CdLS, individuals with FXS showed significantly lower scores on eye contact and less attention to persons but more object attention. An association between social affect and social anxiety in individuals with FXS was found. Regarding developmental trajectories for the FXS group, the higher the age, the higher scores on social responsiveness and social communication skills, and higher scores on anxiety.

Score=12		2	0	2	2	2	1	1	2	
Britton et al., 2020 [103]	Examine the forms and functions of aggressive behavior in boys with FXS as well as the maintainer conditions.	FXS<br>IDD	No	FXS (n=41)<br>Boys<br>11-18 years	Chs	PR	S/VM<br>-FAST [206].	CA	DS<br>EC<br>Y*	Compared to boys with IDD, boys with FXS were significantly more prone to exhibit specific forms of aggressive behaviors as scratching others or biting others. Both groups had similar reinforcements as maintainers of the behaviors.
Score=11		2	0	2	2	0	1	2	2	
Côte et al., 2020 [77]	Examine the specific behavioral problems associated with adaptive functioning in individuals with TS, DS, FXS, and TD.	TS<br>DS<br>FXS<br>TD	No	FXS (n=23)<br>3-30 years<br>8 females/15 males	MC, Socia l medi a	NM	S/VM<br>-The RBS-R [229, 230]<br>-ABC-C [189].<br>-Social Responsiveness Scale [231,232].<br>-The CBCL [218,233].<br>-Brief Symptom Inventory [234].<br>-Adaptive Behavior Assessment-II [235].	SA/SG	DS<br>EC<br>Y*	Compared to the TD group, all clinical groups showed less adaptive behavior, including the social composite, more social issues, higher global scores on autistic questionnaires, and higher scores on lethargy. Specifically, the group with FXS exhibited significantly more irritability, stereotypic behavior, and inappropriate speech than TD individuals. Besides, the groups with FXS and TS showed significantly higher hyperactivity scores, and the FXS and DS group showed greater problems on the SRS questionnaire compared to TD individuals. The FXS and TS group also showed more

										symptoms on the Attention and hyperactivity scale than the TD group. Furthermore, the FXS group showed higher anxiety scores than the TD group, a trend for more serious oppositional problems was also found in the FXS group.
Score=10		2	0	1	2	0	1	2	2	
DaWalt et al., 2021 [78]	Describe developmental trajectories of attention problems, depression/anxiety, and aggressive behavior in individuals with FXS and examine if autism symptoms and parenting features could predict outcomes in these behavioral variables.	No	SG<br>-The CARS [166].	FXS (n=55)<br>6-18 years<br>Boys and girls	FOS	GT	S/VM<br>-The CBCL [196].<br>-Videotaped session to assess mother-child interactions	LS	DS<br>WR	Developmental trajectories of both attention and aggressive problems declined slightly from 6 to 18 years. However, anxious/depressed scores remained stable over the years. Maternal flexibility and autism had impacts on the trajectory of attention problems. Autism comorbidity was associated with fewer declines in attention and aggressive behavior. Higher maternal flexibility was associated with higher declines in attention problems and increases in depression/anxiety scores for their offspring.
Score=11		0	2	2	0	2	1	2	2	

Key for table

NM= Not mentioned

Control group:

-SQ= standardized questionnaire on typically developing children; TD= Typically Developing; FXS+ASD= Fragil X Syndrome and Autism Spectrum Disorder; SMS= Smith Magenis syndrome; DS= Down syndrome; PDD= Pervasive Developmental Disorder; PW= Prader-Willi; WS= Williams syndrome; NSID= Non-specific ID; IDD= Intellectual and Developmental Disability; FAS= Fetal Alcohol Syndrome; DD=Developmental Delay

ASD control:

- S= Statistically; SG= Stablishing Groups; ECI= Excluding comorbid individuals

Recruitment:

-SC= Single clinic or diagnostic centre; MC= multiple clinics or diagnostic centers; Ch= single charity or parent support group; MCh= multiple charities or parent support groups; FOS= Participants recruited from other study; NWM=Newsleters or magazines; IW=Internet websites; R=Registries of families or databases, parent list servers; PA= Public announcements; MAPC= Mail advertisement to past clients; S= Schools;

Syndrome diagnosis:

-PR= Parent Reports; GT=Genetic tests; DPGT= Document Proof of Genetic Testing; CDP= Confirmed Diagnoses by Professional; DPPC= Document Proof of Professional Confirmation

Methodology:

CE=Clinical examination; IQ=Idiosyncratic questionnaire designed to the study; I=Interview; S/VM= Standarized/Validated measures; MM=Multiple methods; CT= Computarized task; MQ= Modified Questionnaire

Developmental factors:

LS= Longitudinal study; MA= Mental Age matched; VMA= Verbal Mental Age matched; NVIQM= Non-verbal IQ matched; VIQM= Verbal IQ matched; EVAM= Expressive vocabulary ability; AS= Accounted by statistical analysis; SG= Stablish groups to compare between ages; MAB=Matched by Adaptive Behavior

Statistics:

DS= Descriptive Statistics/percentages; WC= within syndrome comparative statistics; WR= within syndrome correlations; CFVSF+ASD= Comparisons between groups with FXS and groups with FXS+ASD; CE= Comparative statistics between syndrome and genetically distinct control group. Y/N (*) = sig. diff. found from genetically distinct control / repeated measures/ groups with FXS and groups with FXS+ASD Y/N ES = Effect size reported OR = Odds ratio reported. EME= Estructural model equations.

* A discrepancy was found in this study
